# The Clamping of End-Tidal Carbon Dioxide Does Not Influence Cognitive Function Performance During Moderate Hyperthermia With or Without Skin Temperature Manipulation

**DOI:** 10.3389/fpsyg.2021.788027

**Published:** 2021-12-22

**Authors:** Ricardo Schultz Martins, Phillip J. Wallace, Scott W. Steele, Jake S. Scott, Michael J. Taber, Geoffrey L. Hartley, Stephen S. Cheung

**Affiliations:** ^1^Environmental Ergonomics Laboratory, Department of Kinesiology, Brock University, St. Catharines, ON, Canada; ^2^N^2^M Consulting Inc., St. Catharines, ON, Canada; ^3^Department of Physical and Health Education, Nipissing University, North Bay, ON, Canada

**Keywords:** passive hyperthermia, cognitive function, isocapnia, end-tidal carbon dioxide, clamping, middle cerebral artery velocity, executive function, working memory

## Abstract

Increases in body temperature from heat stress (i.e., hyperthermia) generally impairs cognitive function across a range of domains and complexities, but the relative contribution from skin versus core temperature changes remains unclear. Hyperthermia also elicits a hyperventilatory response that decreases the partial pressure of end-tidal carbon dioxide (P_et_CO_2_) and subsequently cerebral blood flow that may influence cognitive function. We studied the role of skin and core temperature along with P_et_CO_2_ on cognitive function across a range of domains. Eleven males completed a randomized, single-blinded protocol consisting of poikilocapnia (POIKI, no P_et_CO_2_ control) or isocapnia (ISO, P_et_CO_2_ maintained at baseline levels) during passive heating using a water-perfused suit (water temperature ~ 49°C) while middle cerebral artery velocity (MCA_v_) was measured continuously as an index of cerebral blood flow. Cognitive testing was completed at baseline, neutral core-hot skin (37.0 ± 0.2°C-37.4 ± 0.3°C), hot core-hot skin (38.6 ± 0.3°C-38.7 ± 0.2°C), and hot core-cooled skin (38.5 ± 0.3°C-34.7 ± 0.6°C). The cognitive test battery consisted of a detection task (psychomotor processing), 2-back task (working memory), set-shifting and Groton Maze Learning Task (executive function). At hot core-hot skin, poikilocapnia led to significant (both *p* < 0.05) decreases in P_et_CO_2_ (∆−21%) and MCA_v_ (∆−26%) from baseline, while isocapnia clamped P_et_CO_2_ (∆ + 4% from baseline) leading to a significantly (*p* = 0.023) higher MCA_v_ (∆−18% from baseline) compared to poikilocapnia. There were no significant differences in errors made on any task (all *p* > 0.05) irrespective of skin temperature or P_et_CO_2_ manipulation. We conclude that neither skin temperature nor P_et_CO_2_ maintenance significantly alter cognitive function during passive hyperthermia.

## Introduction

Elevations in core temperature (i.e., hyperthermia) increases physiological (e.g., cardiovascular, metabolic), psychological (e.g., thermal discomfort), and neurological (e.g., central processing) strain relative to thermoneutral environments and can lead to impairments in cognitive function (Hancock et al., 2007; Taylor et al., 2016; Schmit et al., 2017). Thermal stress is proposed to induce an inverted-U response with cognitive function, where either heating or cooling beyond a narrow optimal range causes a progressive impairment (Hancock and Vasmatzidis, 1998; Liu et al., 2013). The magnitude of impairment from thermal stress may also be task-dependent, where higher-order cognitive tasks (e.g., executive function, vigilance, working memory) or those requiring motor coordination are more vulnerable to impairment compared to where simple task performance (e.g., psychomotor processing) (Gaoua et al., 2011; Piil et al., 2017).

Cognitive changes may happen well before major changes in whole-body temperature, as changes in skin temperature may alter arousal or distraction, or increase cognitive workload through dual-tasking between performing the cognitive task and increasing effort from monitoring of thermal state and discomfort (Hancock et al., 2007). With heat stress, higher skin temperature with no or just minor elevations in core temperature increased thermal perception and discomfort, along with eliciting more errors (Gaoua et al., 2012) and slower reaction times (Malcolm et al., 2018) on executive function tasks. Skin cooling has been shown to increase variability in detection time during prolonged vigilance tasks almost immediately upon cold water immersion, with no further decrement upon either −0.5°C or − 1.0°C rectal temperature decrease (Cheung et al., 2007). However, high levels of expertise may offset impairment from cold skin or core, as a military simulation of vigilance did not report any decrements over nearly 3 h of cold exposure (0°C air in addition to 5°C water circulating through a water-perfused suit) compared to thermoneutral (22°C air) in trained soldiers (Tikuisis and Keefe, 2007). Overall, the lack of consensus about temperature effects on cognitive function may arise from a lack of control and isolation of skin versus core temperature changes. Based on the potential influence of skin temperature as an independent factor, it is of interest to tease out the relative contribution of skin versus core temperature influences on cognitive function across a range of task domains and complexities.

Another potential mechanism influencing cognitive function during heat stress is changes in arterial CO_2_, as hyperthermic hypocapnia (reduced CO_2_) increases cerebrovascular resistance and decreases cerebral blood flow (CBF; Ainslie et al., 2005; Al-Khazraji et al., 2019). In thermoneutral environments, hyperventilation-induced hypocapnia impaired executive function (Stroop task) through slowed reaction time and increased error rate (Van Diest et al., 2000), while clamping of end-tidal carbon dioxide (P_et_CO_2_) to eucapnic levels during hypoxic stress (isocapnic hypoxia) countered impairments in psychomotor processing reaction time due to hypoxia-induced hypocapnia (Friend et al., 2019). However, countering hyperventilatory-induced hypocapnia during high-intensity exercise (~80% peak maximal oxygen consumption) through the maintenance of P_et_CO_2_ and middle cerebral artery velocity (MCA_v_) did not prevent declines in executive function performance (inhibitory control and spatial working memory; Komiyama et al., 2019). Gibbons et al. (2020) reported that maintaining P_et_CO_2_ at eucapnic levels during passive hyperthermia (∆ + 1.5°C in T_core_) did not affect changes in reaction time on a simple psychomotor and inhibition task (Gibbons et al., 2020). However, it remains unknown if manipulating P_et_CO_2_ influences higher-order cognitive tasks (executive function, working memory) during passive heat exposure.

The purpose of this study was to investigate the role of skin versus core temperature with passive hyperthermia on cognitive function. This was done by eliciting four distinct thermal states: baseline (BASE, neutral core and skin), Neutral Core–Hot Skin (NC-HS), Hot Core–Hot Skin (HC-HS), and Hot Core–Cooled Skin (HC-CS). To isolate the potential confounding role of end-tidal carbon dioxide due to hyperthermic hyperventilation, trials were performed under poikilocapnia (no P_et_CO_2_ manipulation) or with isocapnia (maintenance of P_et_CO_2_ at eucapnic levels). We hypothesized that (i) cognitive performance would be impaired with moderate hyperthermia and predominantly driven by skin rather than core temperature, (ii) cognitive decrements would be greater in poikilocapnia than isocapnia due to a hyperthermia-induced hypocapnia.

## Materials and Methods

The experimental protocol was approved by the Bioscience Research Ethics Board at Brock University (REB 17–385) and conformed to the latest revision of the *Declaration of Helsinki*. Eleven healthy male volunteers (for participant characteristics see Table 1), who were free from cardiovascular, respiratory, and neurological disorders were recruited from the university and community population; all participants were screened by a physician and then provided informed written consent.

**Table 1:** The mean (± SD) participant (*n* = 11) characteristics collected during the preliminary assessment.

Characteristic	Results (mean (± SD))
Age (years)	23 ± 2.4
Mass (kg)	76.2 ± 9.9
Height (cm)	177.5 ± 5.8
Body Fat (%)	11.2 ± 6.4
Peak oxygen consumption (ml·kg^−1^·min^−1^)	47.0 ± 5.7
Cerebrovascular reactivity (cm·s^−1^·mmHg^−1^)	1.33 ± 0.24
Cognitive Failure Questionnaire Score (0–100)	23.0 ± 6.5

### Experimental Design

**Figure 1:**
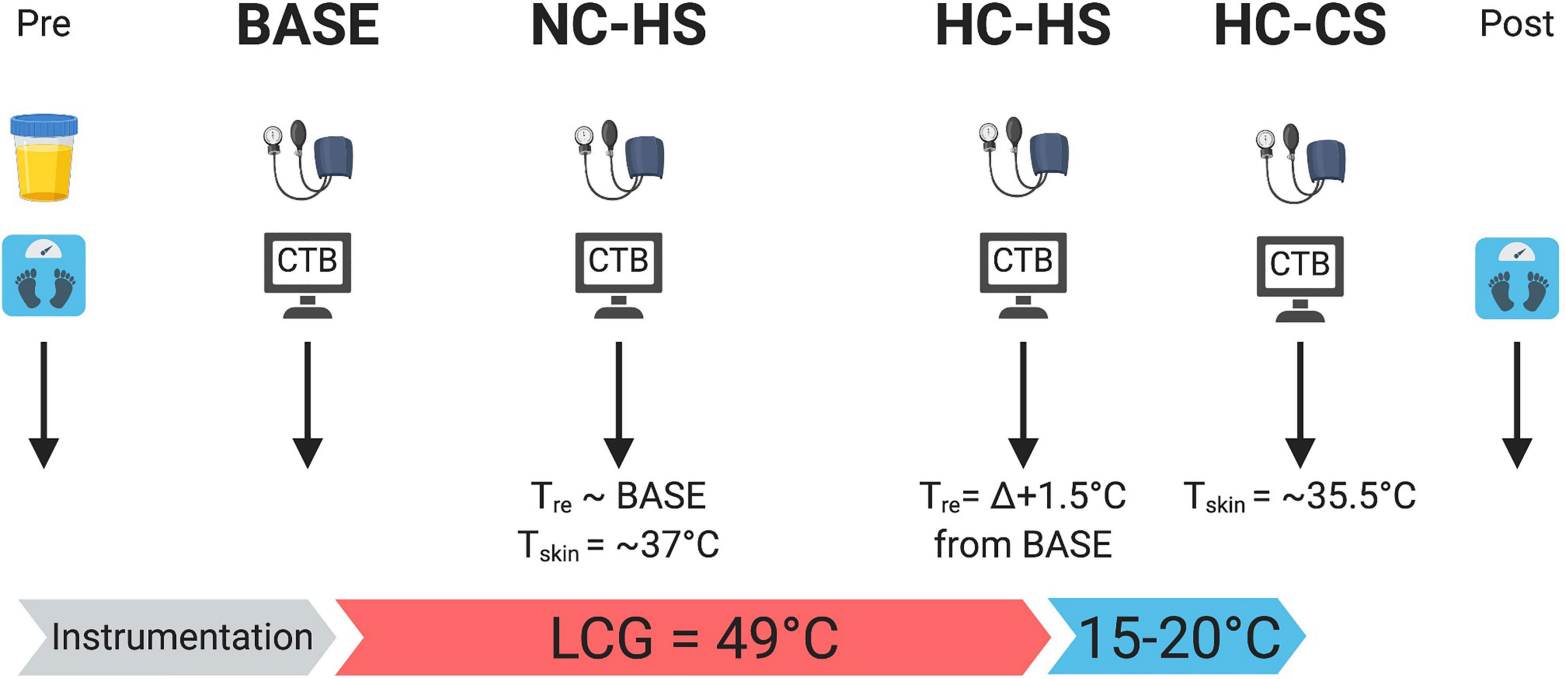
Schematic of experimental protocol. At each experimental time-point, participant’s blood pressure was measured followed by performing the CTB. Body mass was recorded pre and post trial. Urine specific gravity was measured pre trial. Created with BioRender.com.

The experiment implemented a randomized crossover design consisting of a familiarization trial and 2 experimental trials (Figure 1, see details below). Experimental trials were separated by at least one week to reduce the potential for heat acclimation and were performed at the same time of day for each participant to control for circadian fluctuations in core temperature. Participants were instructed to avoid vigorous exercise and alcohol consumption for 24 h, caffeine for 12 h prior to each experimental session, and to follow their typical meal and hydration practices 24 h prior to each session. Randomization was performed online (random.org) and participants were blinded to the trial type being performed.

### Familiarization Trial

Upon arrival to the laboratory, anthropometric measurements (height, mass) and body fat percentage (calculated using the 7-site skinfold technique) were recorded. Participants then completed the Cognitive Failure Questionnaire, which is a 25-item questionnaire that is a self-evaluative measure of general fluid intelligence and related to four factors of absentmindness (memory, distractibility, blunders, and names; Broadbent et al., 1982) where total scores can range between 0 to 100 and the average score is between 19 and 35. Participants were excluded from the study if their score was ≥45, as this score indicates considerable difficulties in completing tasks that require vigilance and may be easily distracted, which could potentially influence the cognitive function data. Of 12 participants initially recruited, one was excluded based on Cognitive Failure Questionnaire score and did not perform the experimental assessments. Next, cerebrovascular reactivity to CO_2_ was assessed through using a custom-built end-tidal forcing system. The cerebrovascular reactivity assessment protocol comprised a 5-min baseline (eucapnia), a subsequent 5-min at hypercapnia (+10 mmHg from eucapnia) followed by 5-min at hypocapnia (−10 mmHg from eucapnia), where reactivity was determined by the change in CBF per change in P_et_CO_2_. Next, participants performed a cognitive test battery (CTB; see details below) in a thermoneutral environment (~22°C, 30% RH) for a total of 3 times to reduce the possibility of a learning effect from repeated exposures (Wallace et al., 2017, 2021). Lastly, an incremental test to exhaustion was performed on a cycle ergometer (Velotron, RacerMate Inc., United States) to determine peak oxygen consumption. The test began with a standardized 5-min warm-up at 100 W, followed by workload increase of 25 W each minute until exhaustion. Peak oxygen consumption was defined as the highest 30-s value measured breath by breath from expired gases collected through a soft silicone facemask connected to an online gas collection system.

### Thermal Manipulations

Upon arrival, participants voided their bladder and nude body mass (kg) was recorded. A sample of the urine was tested for urine specific gravity (PAL-10S, Atago, Japan) to determine hydration status. Participants were considered euhydrated if USG was ≤1.020, or else the test was rescheduled (ultimately, no trials were rescheduled from hypohydration). Participants were then instrumented and fitted with a two-piece liquid conditioning garment (BCS 4 Cooling System, Med Eng, Canada) consisting of 1/8″ diameter Tygon tubing sewn into a stretchable jacket and pant; the head, hands and feet uncovered. Participants then rested in a semi-recumbent seated position (~5-min) and a baseline measure of P_et_CO_2_ was recorded. Next participants performed the BASE measure of the CTB (see details below) in a thermoneutral room (~22°C, 30% RH) with no temperature manipulation. Following BASE, participants were fitted with a polyvinyl rain suit and thermal blanket over the liquid conditioning garment with the hands and head uncovered to minimize evaporative heat loss for the rest of the heating protocol.

**Figure 2:**
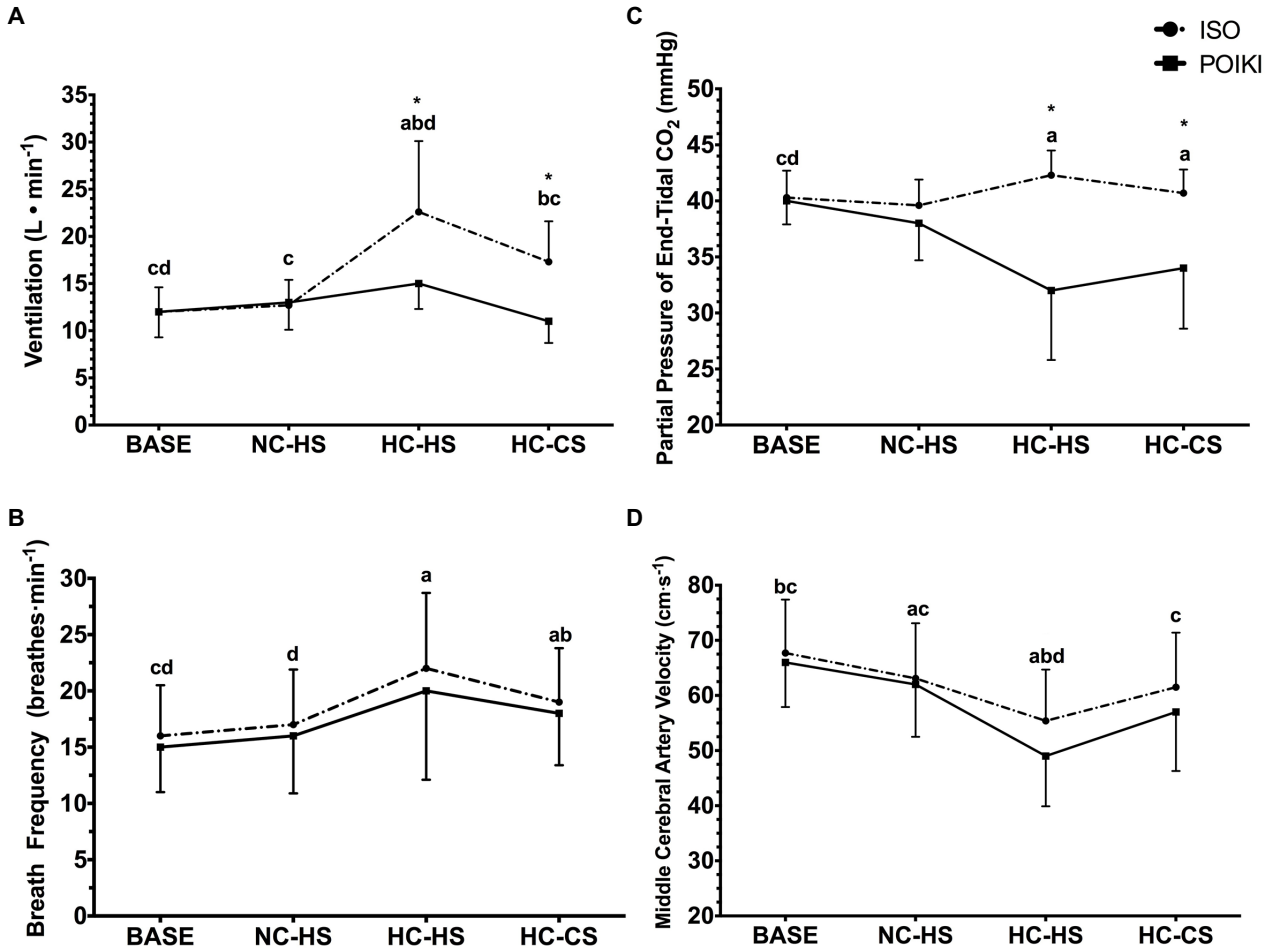
Ventilation [ ${\dot{V}}_{I};$ Panel **(A)**], Breath Frequency [ ${\dot{f}}_{br};$ Panel **(B)**], Partial Pressure of End Tidal Carbon Dioxide [P_et_CO_2_; Panel **(C)**], and Middle Cerebral Artery Velocity [MCA_v_; Panel **(D)**] responses (presented as mean ± SD) for the four experimental time-points. For significant time-point effects (*p* ≤ 0.05), significant (*p* ≤ 0.05) Bonferroni pairwise comparisons can be interpreted as: a significantly different from BASE, b significantly different from NC-HS, c significantly different from HC-HS, d significantly different from HC-CS. For condition effects, * indicates significant (*p* < 0.05) differences between ISO and POIKI at that specific time-point.

Similar to a previous study (Wallace et al., 2021), four time-points were tested manipulating both physiological and perceptual thermal strain (Figure 2). The first time-point was BASE where no temperature manipulation occurred. Next, to delineate the sensory displeasure of hot skin separate from increases in core temperature, ~49.0°C water was circulated at 2.5 l·min^−1^ through the liquid cooling garment and the next CTB was performed once a mean skin temperature ( ${\bar{T}}_{\mathrm{skin}}$ ) of ~37.0°C was achieved, creating the NC-HS time-point. To test the effects of hyperthermia on cognitive performance, passive heat stress was continued until there was a rise in rectal temperature by ~∆ + 1.5°C creating a HC-HS time-point; a ∆ + 1.5°C change in T_re_ was used to ensure a sufficient thermal strain to induce a hyperventilatory hypocapnia response. Additionally, based on pilot work and unpublished reports, our passive heating protocol has interindividual variability in thermal tolerance >38.5°C and led to alterations in ventilatory breathing patterns causing difficulties to clamp P_et_CO_2_. Therefore, a ∆ + 1.5°C ensured thermal tolerance between all participants and allowed for better control of P_et_CO_2_ using the end-tidal forcing system. Lastly, upon completion of the CTB, to test the effects of hyperthermia without sensory displeasure of hot skin, ~15–20°C water was circulated through the liquid conditioning garment until ${\bar{T}}_{\mathrm{skin}}$ of 35.5°C while minimizing changes in core temperature, creating the HC-CS time-point.

### P_et_CO_2_ Manipulations

To determine the role of P_et_CO_2_ on cognitive function during passive heat stress, participants performed identical experimental trials that differed only based on inspired gas manipulation: i) a poikilocapnic (POIKI) condition where P_et_CO_2_ was recorded but not manipulated; and ii) an isocapnic (ISO) condition where P_et_CO_2_ was clamped to eucapnic levels determined at BASE. Participants were fitted with a soft silicone facemask (Hans Rudolph, United States) attached to a T-shaped two-way non-rebreathing valve connected to an online gas collection system (ML206 Gas Analyzer, AD Instruments; United States). Measures of inspired ventilation ( ${\dot{V}}_{I},$ L·min^−1^) and breathing frequency ( ${\dot{f}}_{br},$ breaths·min^−1^), were measured using a pneumotach. Inspired and expired fractions of CO_2_ were sampled at 1 kHz to determine breath-by-breath P_et_CO_2_ and were sampled through nafion tubing in a desiccant-packed drying chamber at a flow rate of 200 ml·min^−1^. A custom built end-tidal forcing system (for details see: Hartley et al., 2016) was used to manipulate P_et_CO_2_ throughout the experimental trials by manipulating inspired CO_2_ concentration to ‘force’ P_et_CO_2_ towards the desired value. Gas concentrations were controlled through solenoid valves which independently controlled gas flow from cylinders of compressed medical grade breathing air (20.93% oxygen, 0.03% CO_2_, balance nitrogen), and 100% CO_2_. Inspired air volumes were delivered to an air reservoir (~5 l) *via* a humidification chamber (~500 ml). Both temperature and humidity of the gas was regulated to replicate ambient room conditions through the end-tidal forcing system.

As a separate quality control for determining the accuracy and signal drift of our gas analyzer over the course of the experimental duration, CO_2_% and ventilation volume were tested every 60-min over a 180-min period on one participant under thermoneutral conditions using ventilation rates of 10–20 l·min^−1^. The average CO_2_ signal drift was ∆ + 0.01 ± 0.01% CO_2_ compared to room air, ∆–0.01 ± 0.01% CO_2_ compared to the calibration gas, and volume was ∆ + 0.01 ± 0.025 l over the 180-min period. Overall, minimal CO_2_ signal drift over the course of the experimental trials (< 3 h in duration) ensuring accuracy of the end-tidal forcing system in clamping P_et_CO_2_ throughout the experimental protocol.

### Instrumentation

Before the commencement of BASE, participants were instrumented with a flexible thermistor (Mon-A-Therm Core, Mallinkrodt Medical, United States), self-inserted 15 cm beyond the anal sphincter to measure rectal temperature (T_re_) sampled at 4 Hz. Four thermocouples (VC-T-24-190 Omega Environmental Inc., Canada) were used to determine weighted ${\bar{T}}_{\mathrm{skin}}$ at four sites defined as ${\bar{T}}_{\mathrm{skin}}=0.3_{arm}+0.3_{chest}+0.2_{thigh}+0.2_{calf}$ on the right side of the body sampled at 4 Hz. Heart rate was calculated using R-R intervals using a standard three-lead electrocardiogram (MLA2340, AD Instruments; United States). Bi-lateral middle cerebral artery velocity (MCA_v_) was assessed using a 2 MHz pulsed transcranial Doppler ultrasound system (Doppler-Box, Compumedics GmbH, Germany). The probes were positioned over the temporal window and were held in place using a secure and comfortable head frame (M600 Headframe, Spencer Technologies, United States). To identify and optimize signals, positioning of the probes were determined by using techniques described by Willie et al. (2011). Data was visually inspected and removed, *post-hoc*, if significant artifacts were detected (e.g., movement during CTB) or poor signal waveform quality. Systolic blood pressure (SBP) and diastolic blood pressure (DBP) were taken from a manual sphygmomanometer (Aneroid Sphygmomanometer, Welch Allyn Hillrom, United States) on their left arm by the same experienced researcher before the CTB at each time-point. Mean arterial pressure (MAP) was calculated as: MAP=((2*DBP)+SBP)/3 . All physiological data (except MAP) were continuously sampled throughout the experiment and are the average over the course of the entire CTB. Subjective assessments of the environmental conditions were assessed using a 1–4 scale to measure thermal comfort and a 1–7 scale for thermal sensation (Gagge et al., 1967) and were collected upon the completion of the CTB at BASE, NC-HS, HC-HS, and HC-CS.

### Cognitive Assessment

To measure progressive changes in cognitive performance, a ~ 12-15 min customized CTB (CogState, United States) was performed at BASE, NC-HS, HC-HS, and HC-CS. This customized CTB focused on the assessment of executive function and working memory. Executive function is an umbrella term for a collection of higher-order cognitive processes and is the ability to plan and execute behavior while being able to dynamically update goals, store and acquire information in working memory, switch among tasks, inhibit behaviors, process errors and being able to perform these behaviors in changing environments (McCabe et al., 2010). Multiple tested were chosen to encapsulate different components of executive function in order to determine potential task-dependent changes in cognitive function. The CTB consisted of a Groton Maze Learning Task (GMLT), a detection task, a two-back task, a set-shifting task, and a GMLT recall task. The order of the tasks was identical CTB was performed. However, the stimuli in each task was randomized to ensure similar task difficulty each time the CTB was performed. For each task, participants were instructed to respond as quickly and accurately as they could.

The GMLT is a touch screen-based test that measures executive function through error detection and spatial memory. The test consists of a hidden 28-step pathway on a 10 × 10 grid of squares. A blue tile on the top left corner of the screen indicates the starting location while a red circle on the bottom right indicates the finish location. The GMLT is performed five times per test and is randomized and matched for difficulty on every trial assessment to minimize learning effects. Performance changes were measured for the total duration (s) and total number of errors (#) measured during the five-block period and for the last maze (GMLT 5). A GMLT recall test (GLMT–Recall) was performed at the end of each CTB, which required the participant to recall the same hidden pathway from the initial five-block period. The total testing period took ~5–7-min.

A detection task was used to test psychomotor reaction time. A face down card was presented on the screen and the participant was required to press a key as soon as the card was turned over. This process continued until the task is completed with 35 cards being presented with a 2 s interval in between. Performance was measured for speed (mean of the log10 transformed reaction times for correct responses) and for total number of errors (#). The task required ~2-min to complete.

A 2-back test was used to measure of attention and visual working memory. This test required the participant to determine if the card being presented is identical to the card presented two cards ago. There was a total of 48 cards presented and the participant could select either “yes” or “no” for each card. Performance for this task was measured for speed of processing (mean of the log_10_ transformed reaction times for correct responses) and total number of errors (#). The task required ~2-min to complete.

A set-shifting task was used measure of cognitive flexibility. In this test, the participant was asked to answer the question “is this the target card?.” The participant was presented with a playing card in the center of the screen with either the word “number” or “colour” above it and had to select “yes” or “no.” The only feedback presented to the participant was that the next card would not be displayed until the correct response is made. The target card changes throughout the test which could be either from one colour to the other (i.e., from a red target card to a black target card or intra-dimensional shift) or from “colour” to “number” (i.e., from a red target card to a number two target card or extra-dimensional shift). The participant was not told when these changes occurred and needed to re-learn the new target card to continue with the test. Performance was measured based on speed of processing (mean of the log_10_ transformed reaction times for correct responses) and total number of errors (#). The task required ~3-min to complete.

### Statistical Analyses

All continuous variable data are presented as the mean ± SD averaged over each time-point while completing the CTB. All physiological and cognitive variables were analyzed using separate condition (ISO vs. POIKI) x time-point (BASE, NC-HS, HC-HS, HC-CS) repeated measures ANOVAs. If data violated sphericity (Mauchley’s test, *p* < 0.05) the Greenhouse–Geisser was used. A Bonferroni *post-hoc* analysis was used to test significant timepoint main effects. Pre and Post body mass was assessed using a condition (ISO x POIKI) by time (PRE x POST) repeated measures ANOVA. If a significant condition x timepoint interaction was calculation, paired sample *t* tests were used to test the main effects at specific time-points (e.g., BASE vs. BASE) to determine if there was a condition effect. To reduce the likelihood of Type I error due to multiple comparison, the α value was revised based on number comparisons (2, i.e., HC-HS POIKI verus HC-HS ISO), therefore *p* = 0.025 for paired samples *t* test comparisons.

All ordinal data (Thermal Comfort, Thermal Sensation) is presented as the median (quartiles 1 and 3) and were analyzed using separate condition (ISO vs. POIKI) x time-point (BASE, NC-HS, HC-HS, HC-CS) repeated measures ANOVAs, with a Wilcoxon signed-rank test used to compare at specific time points if a condition effect was present. All analyses were performed using IBM SPSS Statistics for Windows (version 26.0; IBM Corp., Armonk, N.Y., United States).

## Results

### Experimental Manipulation

The experimental design was successful in creating four distinct temperature and perceptual time-points (Table 2). There was a time-point effect for T_re_ (Table 2; *p* < 0.001, η_p_^2^ = 1.00), where pairwise comparisons determined T_re_ was significantly higher in the HC-HS and HC-CS compared to BASE and NC-HS (all *p* ≤ 0.001). Pairwise comparisons demonstrated no differences in T_re_ between BASE and NC-HS (*p* = 1.00), but a significant difference compared to either HC-HS and HC-CS (both *p* < 0.05). There was a significant conditon effect (*p* = 0.014, η_p_^2^ = 0.766) and time-point effect for T̅_skin_ (Table 2; *p* < 0.001, η_p_^2^ = 1.00), where ${\bar{T}}_{\mathrm{skin}}$ was significantly different at all time-points (all *p* ≤ 0.001). There was a significant timepoint effect for Thermal Comfort (*p* ≤ 0.001, η_p_^2^ = 1.00); where NC-HS (*p* = 0.001) HC-HS (*p* ≤ 0.001), but not HC-CS (*p* = 0.057) were different from BASE; there was no Thermal Comfort difference between NC-HS and HC-CS (*p* = 0.296). Additionally, there was a significant time-point effect for Thermal Sensation (*p* ≤ 0.001, η_p_^2^ = 1.00), where pairwise comparisons demonstrate differences between each time-point (all *p* < 0.05).

**Table 2:** Physiological (presented as mean ± SD) and perceptual (presented as quartiles 1 and 3) for the four experimental time-points.

Variable	BASE	NC-HS	HC-HS	HC-CS
**Core Temperature (°C) ^†^**				
ISO	37.0 ± 0.4	37.0 ± 0.3^cd^	38.5 ± 0.3^ab^	38.3 ± 0.6^ab^
POIKI	37.1 ± 0.4	37.0 ± 0.2^cd^	38.6 ± 0.4 ^ab^	38.4 ± 0.3 ^ab^
**Skin Temperature (°C) ^† #^**				
ISO	32.4 ± 0.4	37.4 ± 0.2^acd^	38.6 ± 0.3^abd^	34.6 ± 0.4^abc^
POIKI	32.7 ± 0.5	37.6 ± 0.4^acd^	38.7 ± 0.2^abd^	35.0 ± 0.6^abc^
**Thermal Comfort (1–4) ^†^**				
ISO	1 (1–1)	2 (2−2)^acd^	4 (3–4)^abd^	1 (1–2)^c^
POIKI	1 (1–1)	2 (2–3)^acd^	4 (3–4)^abd^	2 (1–2)^c^
**Thermal Sensation (1–7) ^†^**				
ISO	4 (3–4)	6 (5–6)^ab^	7 (6–7)^abd^	3 (3–4)^ac^
POIKI	4 (3–4)	6 (5–6)^ab^	7 (6–7)^abd^	3 (2–4)^ac^

^†^ *indicates a significant time-point effect (p ≤ 0.05), where significant (p ≤ 0.05) Bonferroni pairwise comparisons can be interpreted as:*

^a^*significantly different from BASE*.

^b^*significantly different from NC-HS*.

c *significantly different from HC-HS*.

^d^*significantly different from HC-CS*.

^#^*indicates a significant trial effect between ISO and POIKI*.

### Hydration and Body Mass

Urine specific gravity prior to the experimental trials was <1.020 and was not different between conditions (*p* = 0.138, ISO: 1.008 ± 0.006, POIKI: 1.011 ± 0.006). There was a significant time-point effect (*p* ≤ 0.001) for body mass from PRE (ISO: 76.2 ± 10.1 kg, POIKI: 76.2 ± 9.9 kg) to POST (ISO: 74.5 ± 10.2 kg ~ −2.0% loss, POIKI: 74.7 ± 10.1 kg, ~ −2.0% loss) with no differences between conditions (*p* = 0.640).

### Respiratory Responses

There was a significant time-point effect for ${\dot{V}}_{I}$ (*p* ≤ 0.001, η_p_^2^ = 0.999), condition (*p* = 0.005, η_p_^2^ = 0.894), and condition x time-point interaction (*p* = 0.001, η_p_^2^ = 0.964; Figure 2A). Pairwise comparisons revealed a hyperthermia-induced hyperventilatory response where ${\dot{V}}_{I}$ significantly increased from BASE in both HC-HS (*p* = 0.002) and HC-CS (*p* = 0.034). HC-HS was significantly higher than all other time-points (all *p* < 0.05), while there were no differences between NC-HS and BASE (*p* = 0.961) or NC-HS and HC-CS (*p* = 0.105). Paired samples *t*-tests indicated a significant difference in ${\dot{V}}_{I}\;$ between ISO and POIKI at HC-HS (ISO: 22.6 ± 7.5 l·min^−1^, POIKI: 15.3 ± 2.7 l·min^−1^, *p* = 0.005) and HC-CS (ISO: 17.3 ± 4.3 l·min^−1^, POIKI: 11.2 ± 2.3 l·min^−1^, *p* = 0.002). Similarly, ${\dot{f}}_{br}$ demonstrated a significant time-point (*p* = 0.007, η_p_^2^ = 0.853) and condition (*p* = 0.03, η_p_^2^ = 0.628) effect, with a non-significant condition × time-point effect (*p* = 0.619, η_p_^2^ = 0.089; Figure 2B). Pairwise comparisons revealed that compared to BASE, ${\dot{f}}_{br}$ was significantly higher at HC-HS (*p* = 0.021) and HC-CS (*p* ≤ 0.001). Furthermore, HC-CS was significantly higher than NC-HS (*p* = 0.006), with no other significant differences between conditions (all *p* > 0.05).

There was a significant condition (*p* = 0.002, η_p_^2^ = 0.952), time-point (*p* = 0.002, η_p_^2^ = 0.985), and condition x time-point interaction (*p* ≤ 0.001, η_p_^2^ = 0.999) for P_et_CO_2_ (Figure 2C). Pairwise comparisons demonstrated that P_et_CO_2_ was significantly different from BASE at HC-HS (*p* = 0.01) and HC-CS (*p* = 0.022), but not NC-CS (*p* = 0.09). Paired samples t-tests demonstrated there was a significant difference between ISO and POIKI at HC-HS (ISO: 42.3 ± 2.2 mmHg, POIKI: 31.7 ± 6.2 mmHg, *p* = 0.001) and HC-CS (ISO: 40.7 ± 2.1 mmHg, POIKI: 34.2 ± 5.4 mmHg, *p* = 0.004), but not BASE (ISO: 40.3 ± 2.4 mmHg, POIKI: 39.9 ± 2.1 mmHg, *p* = 0.515), or NC-CS (ISO: 39.6 ± 2.3 mmHg, POIKI: 37.9 ± 3.3 mmHg, *p* = 0.203). At HC-HS, the relative change from BASE was ~ + 4.0% in ISO and ~ −26.0% in POIKI.

### Cerebral Hemodynamic Responses

Due to technical issues, data for MCA_v_ was limited to *n* = 8. Within this reduced dataset, there was a significant time-point effect (*p* = 0.006, η_p_^2^ = 0.907), condition (*p* = 0.013, η_p_^2^ = 0.790), but not condition x time-point interaction (*p* = 0.493, η_p_^2^ = 0.198) for MCA_v_ (Figure 2D). Pairwise comparisons demonstrated that, compared to BASE, MCA_v_ was significantly different at NC-HS (*p* = 0.014) and HC-HS (*p* = 0.003), but not HC-CS (*p* = 0.216). HC-HS was significantly lower than all other time-points (all *p* < 0.05). NC-HS was not significantly different than HC-CS (*p* = 1.000). Values for MCA_v_ were BASE (ISO: 67.7 ± 9.7 cm·s^−1^, POIKI: 66.3 ± 8.1 cm·s^−1^), NC-HS (ISO: 63.1 ± 10.0 cm·s^−1^, POIKI: 61.5 ± 9.5 cm·s^−1^), HC-HS (ISO: 55.4 ± 9.3 cm·s^−1^, POIKI: 48.6 ± 9.1 cm·s^−1^), and HC-CS (ISO: 61.0 ± 10.4 cm·s^−1^, POIKI: 57.0 ± 10.7 cm·s^−1^).

### Cardiovascular Responses

**Figure 3:**
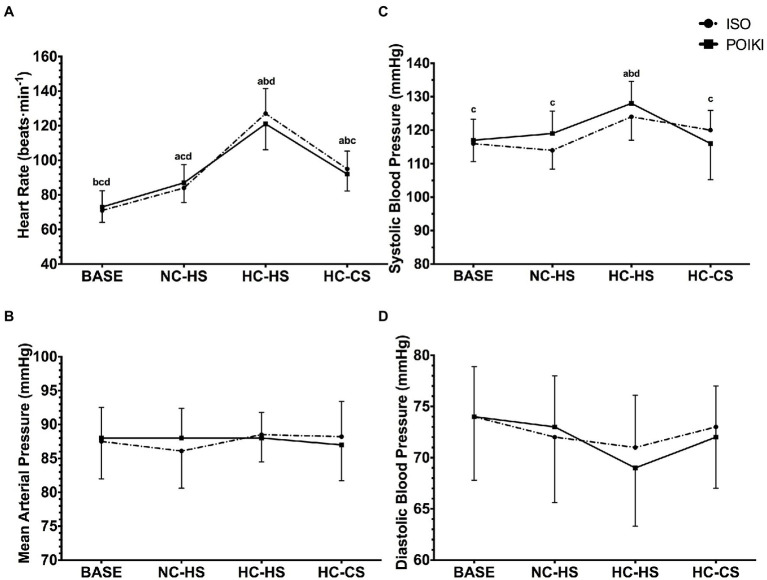
Heart rate Panel **(A)** and Blood Pressure (Mean Arterial Pressure Panel **(B)**, Systolic Blood Pressure Panel **(C)**, and Diastolic Blood Pressure Panel **(D)**) responses (presented as mean ± SD) for the four experimental time-points. For significant time-point effects (*p* ≤ 0.05), significant (*p* ≤ 0.05) Bonferroni pairwise comparisons can be interpreted as: a significantly different from BASE, b significantly different from NC-HS, c significantly different from HC-HS, d significantly different from HC-CS.

Due to poor signal quality of electrocardiogram, one participant’s data was removed from the heart rate analyses (*n* = 10). A significant time-point effect was seen for heart rate (Figure 3A; *p* < 0.001, η_p_^2^ = 1.00) with no condition (*p* = 0.512, η_p_^2^ = 0.094) or interaction (*p* = 0.062, η_p_^2^ = 0.600). Pairwise comparisons demonstrated that heart rate was significantly different from each other at all time-points (all *p* < 0.05). There was no condition (*p* = 0.759, η_p_^2^ = 0.059), time-point (*p* = 0.473, η_p_^2^ = 0.214), or condition x time-point interaction (*p* = 0.264, η_p_^2^ = 0.331) for MAP (Figure 3B). There was a significant time-point effect for SBP (Figure 3C, *p* ≤ 0.001, η_p_^2^ = 1.00), with no condition (*p* = 0.322, η_p_^2^ = 0.157) or condition x time-point interaction (*p* = 0.06, η_p_^2^ = 0.589). Pairwise comparisons revealed SBP was significantly higher at HC-HS compared to all other time-point (*p* ≤ 0.001), while there were no differences between the other time-point (all *p* = 1.000). For DBP (Figure 3D), the time-point effect approached significance (*p* = 0.052, η_p_^2^ = 0.769), however pairwise comparisons demonstrated no differences between any time-point (all *p* > 0.05). There was no condition (*p* = 0.738, η_p_^2^ = 0.061) or condition × time-point interaction (*p* = 0.268, η_p_^2^ = 0.318) for DBP.

### Cognitive Performance

#### GMLT

For the learning portion of the GMLT (i.e., GMLT-1 through 5), there was a significant time-point effect for duration (*p* = 0.017, η_p_^2^ = 0.780), however pairwise comparisons revealed no difference between any time-point (all *p* > 0.05, Table 3A). There was no time-point (*p* = 0.854, η_p_^2^ = 0.094) effect for number of errors made on the learning portion of the GMLT (Table 3A). For GMLT-5 duration (Table 3B), there was a time-point effect (*p* = 0.046, η_p_^2^ = 0.650), however pairwise comparisons revealed no differences between specific time-points (all *p* > 0.05). There was no condition or condition x time-point interaction (both *p* > 0.05). For GMLT-5 errors (Table 3B), there was no time-point (*p* = 0.861, η_p_^2^ = 0.092) or condition x time-point interaction (*p* = 0.981, η_p_^2^ = 0.059) but there was a condition effect (*p* = 0.037, η_p_^2^ = 0.585). For the GMLT-Recall (Table 3C), there were no condition, time-point, or condition x time-point interaction for both duration (all *p* > 0.05) and # of errors made (all *p* > 0.05).

**Table 3:** Cognitive responses (presented as mean ± SD) for the four experimental time-points.

Variable	BASE	NC-HS	HC-HS	HC-NS
**A**				
**GMLT Learning Errors Made (#)**				
ISO	29.0 ± 6.0	30.0 ± 10.0	29.0 ± 11.0	31.0 ± 9.0
POIKI	27.0 ± 9.0	28.0 ± 8.0	26.0 ± 10.0	26.0 ± 6.0
**GMLT Learning Duration (s) †**				
ISO	127.6 ± 29.9	126.6 ± 22.9	111.5 ± 15.7	118.2 ± 15.4
POIKI	122.2 ± 20.8	120.1 ± 19.7	108.4 ± 17.4	114.7 ± 14.5
**B**				
**GMLT-5 Errors Made (#) ^#^**				
ISO	3.0 ± 2.0	3.0 ± 2.0	3.0 ± 2.0	3.0 ± 3.0
POIKI	3.0 ± 1.0	2.0 ± 1.0	2.0 ± 2.0	2.0 ± 1.0
**GMLT-5 Duration (s) ^†^**				
ISO	17.5 ± 5.2	20.2 ± 4.3	17.6 ± 3.0	16.6 ± 2.7
POIKI	18.0 ± 3.3	17.2 ± 3.4	15.7 ± 3.8	15.9 ± 3.2
**C**				
**GMLT-Recall Errors Made (#) †**				
ISO	2.0 ± 1.0	4.0 ± 2.0	3.0 ± 2.0	3.0 ± 2.0
POIKI	2.0 ± 1.0	2.0 ± 2.0	2.0 ± 2.0	2.0 ± 2.0
**GMLT-Recall Duration (s)**				
ISO	18.0 ± 5.2	19.1 ± 5.3	16.8 ± 2.1	17.7 ± 3.7
POIKI	16.7 ± 3.4	17.6 ± 3.5	16.6 ± 4.0	16.7 ± 1.9
**D**				
**Set-Shifting Task Errors Made (#)**				
ISO	20.0 ± 7.0	22.0 ± 8.0	21.0 ± 10.0	21.0 ± 9.0
POIKI	20.0 ± 8.0	20.0 ± 10.0	23.0 ± 8.0	23.0 ± 10.0
**Set-Shifting Task Speed (log10) ^†^**				
ISO	2.35 ± 0.13^c^	2.34 ± 0.12^c^	2.28 ± 0.12^bc^	2.29 ± 0.11
POIKI	2.34 ± 0.14^c^	2.35 ± 0.13^c^	2.27 ± 0.14^bc^	2.31 ± 0.15
**E**				
**2-Back Task Errors Made (#) ^#^**				
ISO	2.0 ± 2.0	2.0 ± 2.0	3.0 ± 2.0	3.0 ± 3.0
POIKI	2.0 ± 1.0	1.0 ± 1.0	3.0 ± 2.0	2.0 ± 1.0
**2-Back Task Speed (log10)**				
ISO	2.83 ± 0.08	2.81 ± 0.10	2.79 ± 0.07	2.78 ± 0.09
POIKI	2.81 ± 0.07	2.81 ± 0.06	2.80 ± 0.08	2.29 ± 0.08
**F**				
**Detection Task Speed (log10) ^†^**				
ISO	2.54 ± 0.08^cd^	2.52 ± 0.06^cd^	2.49 ± 0.05^ab^	2.49 ± 0.06^ab^
POIKI	2.52 ± 0.05^cd^	2.51 ± 0.06^cd^	2.48 ± 0.05^ab^	2.49 ± 0.04^ab^

† *indicates a significant time-point effect (p ≤ 0.05), where significant (p = 0.05) Bonferroni pairwise comparisons can be interpreted as:*

^a^*significantly different from BASE*.

^b^*significantly different from NC-HS*.

c *significantly different from HC-HS*.

^d^*significantly different from HC-CS*.

# *indicates a significant condition effect between ISO and POIKI*.

#### Set-Shifting Task

There was a significant time-point effect for speed (*p* ≤ 0.001, η_p_^2^ = 0.999), where participants were significantly faster at HC-HS compared to BASE (*p* = 0.005), and NC-HS (*p* = 0.008) but not HC-CS (*p* = 0.125; Table 3D). There were no differences between BASE and HC-CS (*p* = 0.055) for set-shifting speed. There was no condition (*p* = 0.901, η_p_^2^ = 0.052) or condition x time-point interaction (*p* = 0.675, η_p_^2^ = 0.142) for set-shifting speed. There was no condition, time-point, or condition x time-point interaction (all *p* > 0.05) for # of errors made on the set-shifting task (Table 3D).

#### 2-Back Task

There was no condition (*p* = 0.896, η_p_^2^ = 0.052), time-point (*p* = 0.061, η_p_^2^ = 0.604), or condition x time-point interaction (*p* = 0.257, η_p_^2^ = 0.337) for speed (Table 3E). There were no time-point (*p* = 0.155, η_p_^2^ = 0.437), or condition x time-point interaction (*p* = 0.538, η_p_^2^ = 0.188), with a condition (*p* = 0.025, η_p_^2^ = 0.660) effect for # of errors on the 2-Back Task (Table 3E).

#### Detection Task

There was a significant time-point effect for Detection Task speed (*p* ≤ 0.001, η_p_^2^ = 1.00), where participants were significantly faster at HC-HS and HC-CS compared to BASE (*p* = 0.008 and 0.039 respectively) and NC-HS (*p* = 0.005 and 0.021 respectively; Table 3F). There was no condition (*p* = 0.190, η_p_^2^ = 0.247) or condition x time-point interaction (*p* = 0.712, η_p_^2^ = 0.131) for Detection Task speed.

## Discussion

This study tested the effect of changes in skin temperature, core temperature, and end-tidal CO_2_ (P_et_CO_2_) on executive function, working memory, and psychomotor processing. We hypothesized that cognitive performance would decrease with an increase in skin temperature and thermal discomfort, before significant core temperature elevation with continued decrements with increase in core temperature. Additionally, we hypothesized that maintaining P_et_CO_2_ and cerebral blood flow would restore some of the heat stress-induced cognitive impairment. Using our test protocol, we demonstrated that neither changes in skin nor core temperature impaired accuracy on any measured cognitive variables. However, hyperthermia (independent of skin temperature) led to faster psychomotor processing, with decreased reaction time on both the detection and set-shifting task. Furthermore, although P_et_CO_2_ was successfully clamped in ISO to slightly above BASE eucapnic levels (∆ + 2 mmHg), we found no benefit from isocapnia and partial restoration of MCA_v_ on cognitive performance throughout our heating protocol.

While many studies report an impairment in cognitive function with whole-body hyperthermia, this finding is not universal. Recent studies with passive or active hyperthermia to ∆ + 1.3–2.0°C in core temperature have not led to decrements in errors for executive function, working memory, or visual perception performance (Schlader et al., 2013; Wallace et al., 2017, 2021; van den Heuvel et al., 2017). Similarly, we found no significant increases in errors made on any of the CTB tasks, but instead significantly faster reaction times on the detection task (psychomotor processing) and set-shifting task (executive function, inhibitory control). Our target hyperthermia of +1.5°C T_re_ was chosen as a compromise between being high enough to elicit hyperthermic hyperventilation versus sufficient capacity to control P_et_CO_2_ with our end-tidal forcing system. It is possible that a core temperature threshold of ≥39°C is needed before cognitive impairments occur (Schmit et al., 2017), such that the absolute thermal strain (T_re_ ~ 38.5–38.6°C) in our, and other null-finding studies, may have been insufficient. It is also possible that individual variability in thermal perception and tolerance may exist due to aerobic fitness and heat acclimation status, body composition, and neurotransmitter concentrations (e.g., dopamine; Cheung and Sleivert, 2004a), which may have influenced cognitive performance during heat stress. Future work is needed to determine the core temperature threshold for hyperthermic impairment and individual factors influencing cognitive function under thermal strain.

Thermal displeasure from sudden alterations in ${\bar{T}}_{\mathrm{skin}}$ can impair cognitive function independent of core temperature changes, with skin cooling the primary driver for vigilance impairments compared to core cooling (Cheung et al., 2007). With heat stress, Gaoua et al. (2012) proposed that an increase in ${\bar{T}}_{\mathrm{skin}}$ can lead to a speed-accuracy trade-off in complex tasks with a faster response time and higher error incidence. Our study aimed to dissociate skin from core temperature effects by targeting testing at timepoints where skin temperature sharply deviated from core temperature (NC-HS and HC-CS). However, an increase in skin temperature by ~5°C and thermal comfort to ‘slightly uncomfortable’ (NC-HS condition) did not affect performance of complex cognitive tasks, nor did the skin cooling of ~4°C and dropping of thermal comfort back to baseline levels of “comfortable” (HC-CS) compared to the typically-studied thermal state of neutral core and skin (Base) or hot core and hot skin (HC-HS, thermal comfort “very uncomfortable”). These results are similar to previous findings testing executive function (inhibitory control) performance where there were no differences in task performance or P300 amplitude between hyperthermia (~∆ + 1.3°C) and hyperthermia with face cooling despite improvements in thermal comfort (Shibasaki et al., 2017). These studies contrast with our previous research using a similar skin/core dissociation protocol to study neuromuscular function, where we have shown that skin temperature is the dominant driver of isokinetic maximal force production with knee extension (Cheung and Sleivert, 2004b) and electromyographic amplitude during a dynamic position task (Coletta et al., 2018). Similarly, regional surface cooling with neck cold collars improved thermal comfort and were sufficient to increase voluntary exercise capacity in hot environments despite no core temperature changes (Tyler and Sunderland, 2011). Overall our data suggests that, unlike in the physiological realm, thermal perception changes driven by skin temperature do not impact cognitive function.

Carbon dioxide levels have a primary influence on CBF (Ainslie et al., 2005) and in physiological systems such as neuromuscular capacity in thermoneutral and hot environments (Ross et al., 2012; Hartley et al., 2016), which may have extended into a role for P_et_CO_2_ changes during passive heating on cognitive function. The ISO condition, which controlled P_et_CO_2_ to baseline levels throughout heating despite perturbations of skin or core temperature, demonstrated similar increases in psychomotor processing and executive function (set-shifting) reaction time without a speed accuracy trade-off with moderate hyperthermia (irrespective of skin temperature) as the POIKI condition with no differences between other tasks. These findings demonstrate that changes in P_et_CO_2_ or MCA_v_ did not alter cognitive performance at moderate hyperthermia levels, and is in line with recent work demonstrating that restoration of P_et_CO_2_. Additionally, MCA_v_ did not counter the decline in executive function (inhibitory control and spatial working memory) performance during high-intensity exercise (Komiyama et al., 2019) or psychomotor processing during passive hyperthermia at sea-level and altitude (Gibbons et al., 2020). Similar to our previous investigations using active hyperthermia (Wallace et al., 2017), we found no decrements in accuracy on executive function (inhibitory control, spatial working memory) or working memory task performance with a ∆ + 1.5°C increase in T_re_. However, in the current study, we did demonstrate an increased psychomotor speed with hyperthermia (irrespective of skin temperature) indicating faster encoding which may have influenced higher-order cognitive function (Racinais et al., 2008). As P_et_CO_2_ has been demonstrated to improve neuromuscular capacity, future studies should determine if P_et_CO_2_ plays a role in complex motor based cognitive tasks (Piil et al., 2017).

Cerebral blood flow is tightly regulated by arterial CO_2_, pH, MAP, cerebral metabolism and autonomic nervous system function (for reviews see: Willie et al., 2014; Bain et al., 2015). We showed that moderate hyperthermia (HC-HS, ∆ + 1.5°C in T_re_) conditions led to a hyperventilatory hypocapnic response (~∆−8 mmHg) with a decrease in MCA_v_ by ~26% in POIKI. Although P_et_CO_2_ was successfully ‘clamped’ just above eucapnic levels leading to significantly higher MCA_v_ compared to POIKI, the hyperventilatory response remained and MCA_v_ was still reduced ~18% relative to thermoneutral baseline. These results are in line with previous investigations (Fujii et al., 2008; Brothers et al., 2009; Ross et al., 2012) but are in contrast to studies that successfully restored MCA_v_ to baseline levels (Nelson et al., 2011; Bain et al., 2013). These discrepancies may be due to our ‘clamping’ P_et_CO_2_ continuously throughout heating versus its restoration only upon reaching hyperthermia (Ross et al., 2012; Bain et al., 2013), our moderate versus severe hyperthermia (Ross et al., 2012; Bain et al., 2013), or the semi-recumbent position used in the current study versus supine postures (Nelson et al., 2011; Bain et al., 2013; Favre et al., 2020). Additionally, ISO demonstrated an exacerbated response during both HC-HS and HC-CS time-points compared to POIKI. The response likely occurred due to the addition of inspiratory CO_2_ by the end-tidal forcing system increasing the overall inspiratory volume. Conversely, the trend for a higher (~1–2 breaths·min^−1^) at HC-HS with ISO may indicate that the additional CO_2_ increased minute ventilation by stimulating the respiratory centres in the brain (Nattie, 1999). There is the potential that the additional inspiratory volume may have interfered with neural processing and potentially influencing cognitive function during the ISO condition. However, there were no condition or condition x timepoint interactions between the ISO and POIKI for any of the cognitive variables. However, future research is needed to determine the role of P_et_CO_2_ and ventilation on neural processing (using such tools as electroencephalography) during heat stress. Lastly, this study is limited by not having a measure of intracranial pressure or cerebral perfusion, which are influenced by changes in MAP and vascular resistance (for review see: Numan et al., 2014). Recently, Shoemaker et al., (2019) determined that, under hypercapnia in thermoneutral conditions, CO_2_ is the primary driver for changes in MCA_v_ as opposed to MAP changes that occur during acute mental stress caused by performing a subtraction task. In the current study, it is unlikely that there were differences in cerebral perfusion as MAP remained relatively unchanged throughout heating, as well as participants remained in the semi-recumbent posture throughout heating to reduce the impact of positional changes on MAP and cerebral perfusion (Nelson et al., 2011; Numan et al., 2014).

A strength of our research experiment was the use of an end-tidal forcing system that allowed us to accurately clamp P_et_CO_2_ (~1–4% from BASE) breath-by-breath for the entirety of the ISO trial (Figure 1). This method provides an accurate control of P_et_CO_2_ as an indirect measure of arterial PCO_2_, where P_et_CO_2_ has been demonstrated to accurately estimate arterial PCO_2_ throughout passive hyperthermia up to +2.0°C (Brothers et al., 2009; Ross et al., 2012; Bain et al., 2013)_._ Breath-by-breath clamping of P_et_CO_2_ is more advantageous compared to CO_2_ bolus techniques because, once hypocapnic, the CBF responses is slower to respond to changes in CO_2_ (Poulin et al., 1998; Ross et al., 2012). Therefore, with use of the end-tidal forcing system, we prevented hypocapnia in order to control CBF. A limitation of our study was the use of TCD to measure MCA_v_ as an index representation of CBF, which is a valid relationship if assuming the diameter of the MCA does not change (Ainslie and Hoiland, 2014). Direct measurement of the MCA using magnetic resonance imaging determined that the MCA dilates during 10 min of hypercapnia (~48 mmHg P_et_CO_2_) and constricts during hypocapnia (~31 mmHg P_et_CO_2_) in thermoneutral conditions (Al-Khazraji et al., 2019). We clamped P_et_CO_2_ throughout the ISO trial to BASE levels which may have minimized vasodilation of cerebrovascular vessels. Additionally, we cannot account for local CBF changes in the executive attention network and prefrontal cortex, which are important neural regions for the executive function and working memory during passive hyperthermia (Liu et al., 2013; Qian et al., 2013). Furthermore, a limitation of the study is there was no decrement in cognitive performance with manipulation of skin and core temperature. Future research is needed with a cognitive test battery where cognitive function is impaired in order to determine if P_et_CO_2_ can counter decline in performance. Lastly, the results of this study are limited to males as no females were tested to account for fluctuations in core temperature due to the menstrual cycle. Under thermoneutral conditions, the evidence is mixed on whether there are sex-differences in cerebrovascular reactivity (Favre et al., 2020). Future evidence is needed to determine if there are sex-related differences in cognition, changes in P_et_CO_2_, and CBF regulation during passive heat stress.

In summary, the results from our study showed that an increase in both skin and core temperature and thermal discomfort or reduction in MCA_v_ did not impair cognitive function during a passive heating protocol when learning effect is controlled. However, the results did indicate that hyperthermia decreased reaction time on the detection task as well as the set-shifting task. In addition, our results showed a reduction in MCA_v_ regardless of the maintenance of baseline P_et_CO_2_ during hyperthermia suggesting that hyperthermia-induced changes in CBF may have other controlling factors, such as cardiovascular control or skin temperature. Future research is needed to determine how longer exposure and/or different modes of heat stress and hyperthermia may affect cognition.

## Data Availability Statement

The raw data supporting the conclusions of this article will be made available by the authors, without undue reservation.

## Ethics Statement

The studies involving human participants were reviewed and approved by Brock University Biosciences Research Ethics Board. The patients/participants provided their written informed consent to participate in this study.

## Author Contributions

RM, PW, GH, MT, and SC conceived and designed the study. RM, PW, JS, and SS collected the data. PW, RM, GH, JS, and SS reduced and analysed the data. PW, RM, and SC drafted the manuscript. JS, SS, GH, and MT edited the manuscript. All authors contributed to the article and approved the submitted version.

## Funding

This study was supported by a Discovery grant from the Natural Science and Engineering Research Council (NSERC) of Canada (SSC, 2018–04077). RM was supported through an Ontario Graduate Scholarship and PW was supported through a NSERC Doctoral (PGS D) scholarship.

## Conflict of Interest

MT is employed by N2M Consulting Inc.

The remaining authors declare that the research was conducted in the absence of any commercial or financial relationships that could be construed as a potential conflict of interest.

## Publisher’s Note

All claims expressed in this article are solely those of the authors and do not necessarily represent those of their affiliated organizations, or those of the publisher, the editors and the reviewers. Any product that may be evaluated in this article, or claim that may be made by its manufacturer, is not guaranteed or endorsed by the publisher.

## References

[ref1] Ainslie P. N. Ashmead J. C. Ide K. Morgan B. J. Poulin M. J. (2005). Differential responses to CO_2_ and sympathetic stimulation in the cerebral and femoral circulations in humans: blood flow responses to sympathetic stimulation and CO_2_ in humans. J. Physiol. 566, 613–624. doi: 10.1113/jphysiol.2005.087320, PMID: 15890697 PMC1464750

[ref2] Ainslie P. N. Hoiland R. L. (2014). Transcranial Doppler ultrasound: valid, invalid, or both? J. Appl. Physiol. 117, 1081–1083. doi: 10.1152/japplphysiol.00854.2014, PMID: 25257879

[ref3] Al-Khazraji B. K. Shoemaker L. N. Gati J. S. Szekeres T. Shoemaker J. K. (2019). Reactivity of larger intracranial arteries using 7 T MRI in young adults. J. Cereb. Blood Flow Metab. 39, 1204–1214. doi: 10.1177/0271678X18762880, PMID: 29513623 PMC6668520

[ref40] Bain A. R. Nybo L. Ainslie P. N. (2015). “Cerebral Vascular Control and Metabolism in Heat Stress,” in Comprehensive Physiology, ed. Terjung G. R. (Hoboken, NJ, USA: John Wiley & Sons, Inc.), 1345–1380. doi: 10.1002/cphy.c140066 26140721

[ref4] Bain A. R. Smith K. J. Lewis N. C. Foster G. E. Wildfong K. W. Willie C. K.. (2013). Regional changes in brain blood flow during severe passive hyperthermia: effects of PaCO_2_ and extracranial blood flow. J. Appl. Physiol. 115, 653–659. doi: 10.1152/japplphysiol.00394.2013, PMID: 23823149

[ref5] Broadbent D. E. Cooper P. F. FitzGerald P. Parkes K. R. (1982). The Cognitive Failures Questionnaire (CFQ) and its correlates. Br. J. Clin. Psychol. 21, 1–16. doi: 10.1111/j.2044-8260.1982.tb01421.x, PMID: 7126941

[ref6] Brothers R. M. Wingo J. E. Hubing K. A. Crandall C. G. (2009). The effects of reduced end-tidal carbon dioxide tension on cerebral blood flow during heat stress: cerebrovascular blood flow during heat stress. J. Physiol. 587, 3921–3927. doi: 10.1113/jphysiol.2009.172023, PMID: 19528251 PMC2746619

[ref7] Cheung S. S. Sleivert G. G. (2004a). Multiple triggers for hyperthermic fatigue and exhaustion. Exerc. Sport Sci. Rev. 32, 100–106. doi: 10.1097/00003677-200407000-00005, PMID: 15243205

[ref8] Cheung S. S. Sleivert G. G. (2004b). Lowering of skin temperature decreases isokinetic maximal force production independent of core temperature. Eur. J. Appl. Physiol. 91, 723–728. doi: 10.1007/s00421-004-1062-0, PMID: 15015000

[ref9] Cheung S. S. Westwood D. A. Knox M. K. (2007). Mild body cooling impairs attention via distraction from skin cooling. Ergonomics 50, 275–288. doi: 10.1080/00140130601068683, PMID: 17419159

[ref10] Coletta N. A. Mallette M. M. Gabriel D. A. Tyler C. J. Cheung S. S. (2018). Core and skin temperature influences on the surface electromyographic responses to an isometric force and position task. PLoS One 13:e0195219. doi: 10.1371/journal.pone.0195219, PMID: 29596491 PMC5875857

[ref11] Favre M. E. Lim V. Falvo M. J. Serrador J. M. (2020). Cerebrovascular reactivity and cerebral autoregulation are improved in the supine posture compared to upright in healthy men and women ed. Mogi M. PLOS ONE 15:e0229049. doi: 10.1371/journal.pone.0229049, PMID: 32119678 PMC7051088

[ref12] Friend A. T. Balanos G. M. Lucas S. J. E. (2019). Isolating the independent effects of hypoxia and hyperventilation-induced hypocapnia on cerebral haemodynamics and cognitive function. Exp Physiol EP087602. 104, 1482–1493. doi: 10.1113/EP087602, PMID: 31342596

[ref38] Fujii N. Honda Y. Hayashi K. Kondo N. Koga S. Nishiyasu T. (2008). Effects of chemoreflexes on hyperthermic hyperventilation and cerebral blood velocity in resting heated humans: Hyperthermic hyperpnoea and chemoreflex drive, cerebral circulation. Exp. Physiol. 93, 994–1001. doi: 10.1113/expphysiol.2008.042143, PMID: 18403444

[ref13] Gagge A. P. Stolwijk J. A. J. Hardy J. D. (1967). Comfort and thermal sensations and associated physiological responses at various ambient temperatures. Environ. Res. 1, 1–20. doi: 10.1016/0013-9351(67)90002-3, PMID: 5614624

[ref39] Gaoua N. Grantham J. Racinais S. El Massioui F. (2012). Sensory displeasure reduces complex cognitive performance in the heat. J. Environ. Psychol. 32, 158–163. doi: 10.1016/j.jenvp.2012.01.002, PMID: 21070137

[ref14] Gaoua N. Racinais S. Grantham J. Massioui F. E. (2011). Alterations in cognitive performance during passive hyperthermia are task dependent. Int. J. Hyperth. 27, 1–9. doi: 10.3109/02656736.2010.516305, PMID: 21070137 PMC3082171

[ref15] Gibbons T. D. Tymko M. M. Thomas K. N. Wilson L. C. Stembridge M. Caldwell H. G.. (2020). Global REACH 2018: The influence of acute and chronic hypoxia on cerebral haemodynamics and related functional outcomes during cold and heat stress. J. Physiol. 598, 265–284. doi: 10.1113/JP278917, PMID: 31696936

[ref16] Hancock P. A. Ross J. M. Szalma J. L. (2007). A meta-analysis of performance response Under thermal stressors. Hum Factors J Hum Factors Ergon Soc 49, 851–877. doi: 10.1518/001872007X230226, PMID: 17915603

[ref17] Hancock P. A. Vasmatzidis I. (1998). Human occupational and performance limits under stress: the thermal environment as a prototypical example. Ergonomics 41, 1169–1191. doi: 10.1080/001401398186469, PMID: 9715675

[ref18] Hartley G. L. Watson C. L. Ainslie P. N. Tokuno C. D. Greenway M. J. Gabriel D. A.. (2016). Corticospinal excitability is associated with hypocapnia but not changes in cerebral blood flow: cerebral blood flow and hypocapnia on corticospinal excitability. J. Physiol. 594, 3423–3437. doi: 10.1113/JP271914, PMID: 26836470 PMC4908026

[ref20] Komiyama T. Tanoue Y. Sudo M. Costello J. T. Uehara Y. Higaki Y.. (2019). Cognitive impairment during high-intensity exercise: influence of cerebral blood flow. Med. Sci. Sports Exerc. 1. doi: 10.1249/MSS.0000000000002183 31609297

[ref21] Liu K. Sun G. Li B. Jiang Q. Yang X. Li M.. (2013). The impact of passive hyperthermia on human attention networks: An fMRI study. Behav. Brain Res. 243, 220–230. doi: 10.1016/j.bbr.2013.01.013, PMID: 23333840

[ref41] Malcolm R. A. Cooper S. Folland J. P. Tyler C. J. Sunderland C. (2018). Passive heat exposure alters perception and executive function. Front. Physiol. 9. doi: 10.3389/fphys.2018.00585, PMID: 29887804 PMC5981197

[ref22] McCabe D. P. Roediger H. L. McDaniel M. A. Balota D. A. Hambrick D. Z. (2010). The relationship between working memory capacity and executive functioning: evidence for a common executive attention construct. Neuropsychology 24, 222–243. doi: 10.1037/a0017619, PMID: 20230116 PMC2852635

[ref42] Nattie E. (1999). CO_2_, brainstem chemoreceptors and breathing. Prog. Neurobiol. 59, 299–331. doi: 10.1016/S0301-0082(99)00008-8, PMID: 10501632

[ref43] Nelson M. D. Haykowsky M. J. Stickland M. K. Altamirano-Diaz L. A. Willie C. K. Smith K. J.. (2011). Reductions in cerebral blood flow during passive heat stress in humans: partitioning the mechanisms: Cerebral blood flow during passive heat stress. J. Physiol. 589, 4053–4064. doi: 10.1113/jphysiol.2011.212118, PMID: 21690194 PMC3180002

[ref44] Numan T. Bain A. R. Hoiland R. L. Smirl J. D. Lewis N. C. Ainslie P. N. (2014). Static autoregulation in humans: a review and reanalysis. Med. Eng. Phys. 36, 1487–1495. doi: 10.1016/j.medengphy.2014.08.001, PMID: 25205587

[ref23] Piil J. F. Lundbye-Jensen J. Trangmar S. J. Nybo L. (2017). Performance in complex motor tasks deteriorates in hyperthermic humans. Temperature 4, 420–428. doi: 10.1080/23328940.2017.1368877, PMID: 29435481 PMC5800368

[ref24] Poulin M. J. Liang P.-J. Robbins P. A. (1998). Fast and slow components of cerebral blood flow response to step decreases in end-tidal P_a_CO_2_ in humans. J. Appl. Physiol. 85, 388–397. doi: 10.1152/jappl.1998.85.2.388, PMID: 9688710

[ref25] Qian S. Sun G. Jiang Q. Liu K. Li B. Li M.. (2013). Altered topological patterns of large-scale brain functional networks during passive hyperthermia. Brain Cogn. 83, 121–131. doi: 10.1016/j.bandc.2013.07.013, PMID: 23959081

[ref26] Racinais S. Gaoua N. Grantham J. (2008). Hyperthermia impairs short-term memory and peripheral motor drive transmission. J. Physiol. 586, 4751–4762. doi: 10.1113/jphysiol.2008.157420, PMID: 18703579 PMC2607529

[ref27] Ross E. Z. Cotter J. D. Wilson L. Fan J.-L. Lucas S. J. E. Ainslie P. N. (2012). Cerebrovascular and corticomotor function during progressive passive hyperthermia in humans. J. Appl. Physiol. 112, 748–758. doi: 10.1152/japplphysiol.00988.2011, PMID: 22134692

[ref28] Schlader Z. J. Lucas R. A. I. Pearson J. Crandall C. G. (2013). Hyperthermia does not alter the increase in cerebral perfusion during cognitive activation: cerebral perfusion during cognitive activation. Exp. Physiol. 98, 1597–1607. doi: 10.1113/expphysiol.2013.074104, PMID: 23851918 PMC4961043

[ref29] Schmit C. Hausswirth C. Le Meur Y. Duffield R. (2017). Cognitive functioning and heat strain: performance responses and protective strategies. Sports Med. 47, 1289–1302. doi: 10.1007/s40279-016-0657-z, PMID: 27988874

[ref30] Shibasaki M. Namba M. Oshiro M. Kakigi R. Nakata H. (2017). Suppression of Cognitive Function in Hyperthermia; From the viewpoint of executive and inhibitive cognitive processing. Sci. Rep. 7. doi: 10.1038/srep43528, PMID: 28497797 PMC5353598

[ref46] Shoemaker L. N. Wilson L. C. Lucas S. J. E. Machado L. Cotter J. D. (2019). Cerebrovascular regulation is not blunted during mental stress. Exp. Physiol. 104, 1678–1687. doi: 10.1113/EP087832, PMID: 31465595

[ref31] Taylor L. Watkins S. L. Marshall H. Dascombe B. J. Foster J. (2016). The impact of different environmental conditions on cognitive function: A focused review. Front. Physiol. 6. doi: 10.3389/fphys.2015.00372, PMID: 26779029 PMC4701920

[ref32] Tikuisis P. Keefe A. A. (2007). Effects of cold strain on simulated sentry duty and marksmanship. Aviat. Space Environ. Med. 78, 399–407. 17484343

[ref33] Tyler C. J. Sunderland C. (2011). Neck cooling and running performance in the heat: single versus repeated application. Med. Sci. Sports Exerc. 43, 2388–2395. doi: 10.1249/MSS.0b013e318222ef72, PMID: 21606877

[ref19] van den Heuvel A. M. J. Haberley B. J. Hoyle D. J. R. Taylor N. A. S. Croft R. J. (2017). The independent influences of heat strain and dehydration upon cognition. Eur. J. Appl. Physiol. 117, 1025–1037. doi: 10.1007/s00421-017-3592-2, PMID: 28343279

[ref34] Van Diest I. Stegen K. Van de Woestijne K. P. Schippers N. Van den Bergh O. (2000). Hyperventilation and attention: effects of hypocapnia on performance in a Stroop task. Biol. Psychol. 53, 233–252. doi: 10.1016/S0301-0511(00)00045-4, PMID: 10967234

[ref35] Wallace P. J. Martins R. S. Scott J. S. Steele S. W. Greenway M. Cheung S. S. (2021). The effects of acute dopamine reuptake inhibition on cognitive function during passive hyperthermia. Appl. Physiol. Nutr. Metab. apnm. 46, 511–520. doi: 10.1139/apnm-2020-0869 33232172

[ref36] Wallace P. J. McKinlay B. J. Coletta N. A. Vlaar J. I. Taber M. J. Wilson P. M.. (2017). Effects of motivational self-talk on endurance and cognitive performance in the heat. Med. Sci. Sports Exerc. 49, 191–199. doi: 10.1249/MSS.0000000000001087, PMID: 27580154

[ref37] Willie C. K. Colino F. L. Bailey D. M. Tzeng Y. C. Binsted G. Jones L. W.. (2011). Utility of transcranial Doppler ultrasound for the integrative assessment of cerebrovascular function. J. Neurosci. Methods 196, 221–237. doi: 10.1016/j.jneumeth.2011.01.011, PMID: 21276818

[ref45] Willie C. K. Tzeng Y.-C. Fisher J. A. Ainslie P. N. (2014). Integrative regulation of human brain blood flow: Integrative regulation of human brain blood flow. J. Physiol. 592, 841–859. doi: 10.1113/jphysiol.2013.268953 24396059 PMC3948549

